# Minimally Invasive Management of Bladder Stones in Children

**DOI:** 10.3389/fped.2020.618756

**Published:** 2021-01-26

**Authors:** Ciro Esposito, Giuseppe Autorino, Lorenzo Masieri, Marco Castagnetti, Fulvia Del Conte, Vincenzo Coppola, Mariapina Cerulo, Felice Crocetto, Maria Escolino

**Affiliations:** ^1^Pediatric Surgery Unit, Federico II University of Naples, Naples, Italy; ^2^Pediatric Urology Unit, Meyer Children Hospital, Florence, Italy; ^3^Pediatric Urology Unit, Medical University of Padua, Padua, Italy; ^4^Urology Unit, Federico II University of Naples, Naples, Italy

**Keywords:** bladder stones, children, endoscopy, laser, robotic surgery, stone free rate

## Abstract

**Background:** Bladder stones (BS) are rare in children. Minimally invasive surgery (MIS) seems to be nowadays the procedure of choice to treat pediatric patients with BS. This study aimed to analyze retrospectively our experience with percutaneous cystolithotomy, endourological treatment with Holmium laser and robotic cystolithotomy in children with BS.

**Methods:** We retrospectively analyzed the data of 13 children (eight boys and five girls) with BS who were treated at our centers between July 2013 and July 2020. The patients received three different MIS procedures for stones removal: five underwent robotic cystolithotomy, five underwent endourological treatment and three received percutaneous cystolithotomy (PCCL). We preferentially adopted endourological approach for stones <10 mm, percutaneous approach between 2014 and 2016 and robotic approach since 2016 for larger stones.

**Results:** Mean patients' age at the time of diagnosis was 13 years (range 5–18). Ten/13 patients (76.9%) had primary BS and 3/13 patients (23.1%) had secondary BS. Mean stone size was 18.8 mm (range 7–50). In all cases the stones were removed successfully. One Clavien II post-operative complication occurred following PCCL (33.3%). All the procedures were completed without conversions. Operative time ranged between 40 and 90 min (mean 66) with no significant difference between the three methods (*p* = 0.8). Indwelling bladder catheter duration was significantly longer after PCCL (mean 72 h) compared with robotic and endourological approaches (mean 15.6 h) (*p* = 0.001). Hospitalization was significantly longer after PCCL (mean 7.6 days) compared with the other two approaches (mean 4.7 days) (*p* = 0.001). The endourological approach was the most cost-effective method compared with the other two approaches (*p* = 0.001).

**Conclusions:** Minimally invasive management of bladder stones in children was safe and effective. Endourological management was the most cost-effective method, allowing a shorter hospital stay compared with the other procedures but it was mainly indicated for smaller stones with a diameter < 10 mm. Based upon our preliminary results, robotic surgery seemed to be a feasible treatment option for BS larger than 15–20 mm. It allowed to remove the big stones without crushing them with a safe and easy closure of the bladder wall thanks to the easy suturing provided by the Robot technology.

## Introduction

Urolithiasis in childhood is rare in the developed world, and bladder stones (BS) represent 1 to 5% of all urinary tract stones (1–4). In developed countries the main components of BS are struvite or calcium oxalate dihydrate, while in developing countries the main component is ammonium acid urate (5–7). In the last few years, transurethral lithotripsy has become an alternative method to open cystolithotomy (8–10). However, the adoption of this approach in the pediatric patients is limited by the narrow caliber of the urethra in children (11). For this reason, percutaneous techniques using nephroscope or operative optic were developed, with the aim to remove bladder stones quickly in large fragments (12–15). With the increasing use of percutaneous techniques, an alternative solution to remove BS in children could be performing a percutaneous suprapubic bladder stone removal (16). Percutaneous cystolithotomy (PCCL) has been demonstrated to be adequate, safe and quick to perform in managing BS in children (13, 14). However, the main problem with this technique is the difficult closure of the bladder especially for big stones.

More recently, in case of large BS, robotic surgery has demostrated to be an excellent solution to remove BS safely (17–19). The advantage of robotic management was the possibility to remove the stones through the navel using an endobag, without crushing them. Additionally, using the Robot technology, it was easy to open and to close the bladder thanks to the easy suturing allowed by the robotic endowrist instrumentation.

In this study we retrospectively analyzed the management of BS using minimally invasive surgery (MIS) in our centers comparing the indications and the results of three different techniques.

## Materials and Methods

We retrospectively analyzed the data of 13 children (eight boys and five girls) with BS, who were treated at our centers between July 2013 and July 2020.

**Figure 1:**
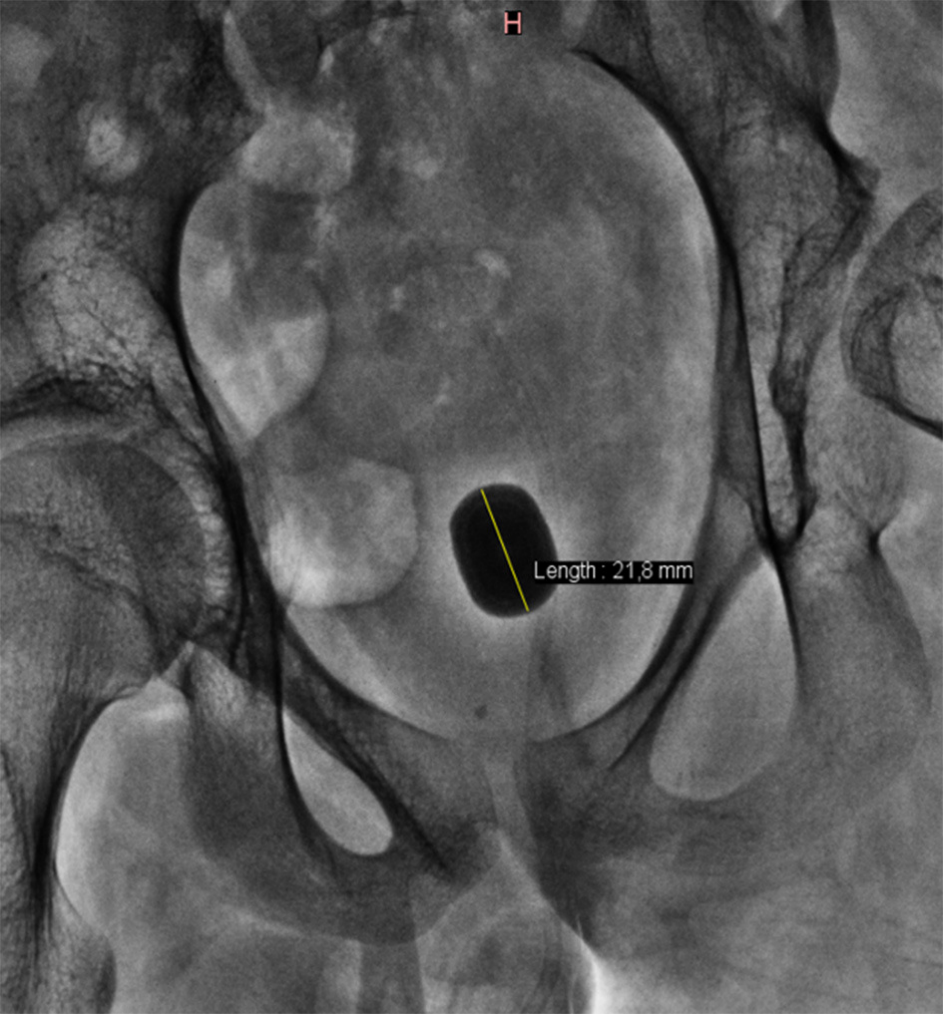
X-ray showing a big bladder stone.

Pre-operative work-up was focused to rule out any possible anatomical malformations of the entire urinary tract and their functional implications. Investigations included ultrasonography and plain abdominal radiograph to measure the stone size (Figure 1). Rarely a computed tomography was performed.

Cystoscopy was always performed pre-operatively. In patients undergoing robotics, we adopted the following protocol of intestinal preparation: simethicone, 15 drops three times per day for 1 week prior to surgery; enemas and minimal-residue diet (low-fiber intake) the day before surgery. All these measures were easily performed by parents at home, without increasing the length of stay. Pre-operative antibiotic prophylaxis was administered either with a broad-spectrum medication or according to the child's specific urine testing.

All patients and their parents signed a specifically formulated informed consent before the procedure. Patients received general anesthesia with oro-tracheal intubation and myorelaxation. A Foley catheter was positioned into the bladder using sterile precautions just before surgery and a nasogastric tube was placed in order to keep the stomach empty during the procedure.

The patients underwent three different MIS procedures for stones removal in our series: five received robotic cystolithotomy, five underwent endourological treatment using Holmium laser *via* transurethral route and three received percutaneous cystolithotomy (PCCL) using MIS suprapubic approach with an operative laparoscope. We preferred endourological approach to treat small stones with a diameter < 10 mm, whereas we preferentially adopted percutaneous approach between 2014 and 2016 and robotic approach since 2016 for larger stones.

From the technical point of view, in the endourological approach, an operative cystoscope with a variable diameter according with the patient's age was adopted and the stones were dusted using a Holmium laser fiber with a core size of 270 μm or less. In the percutaneous approach, a nephroscope or a laparoscopic optic with operative working channel was adopted and the stones were broken and removed in small fragments. According to our protocol, we kept the bladder catheter longer time in patients receiving PCCL than those undergoing the other procedures, in order to allow a safe closure of the bladder and avoid complications.

**Figure 2:**
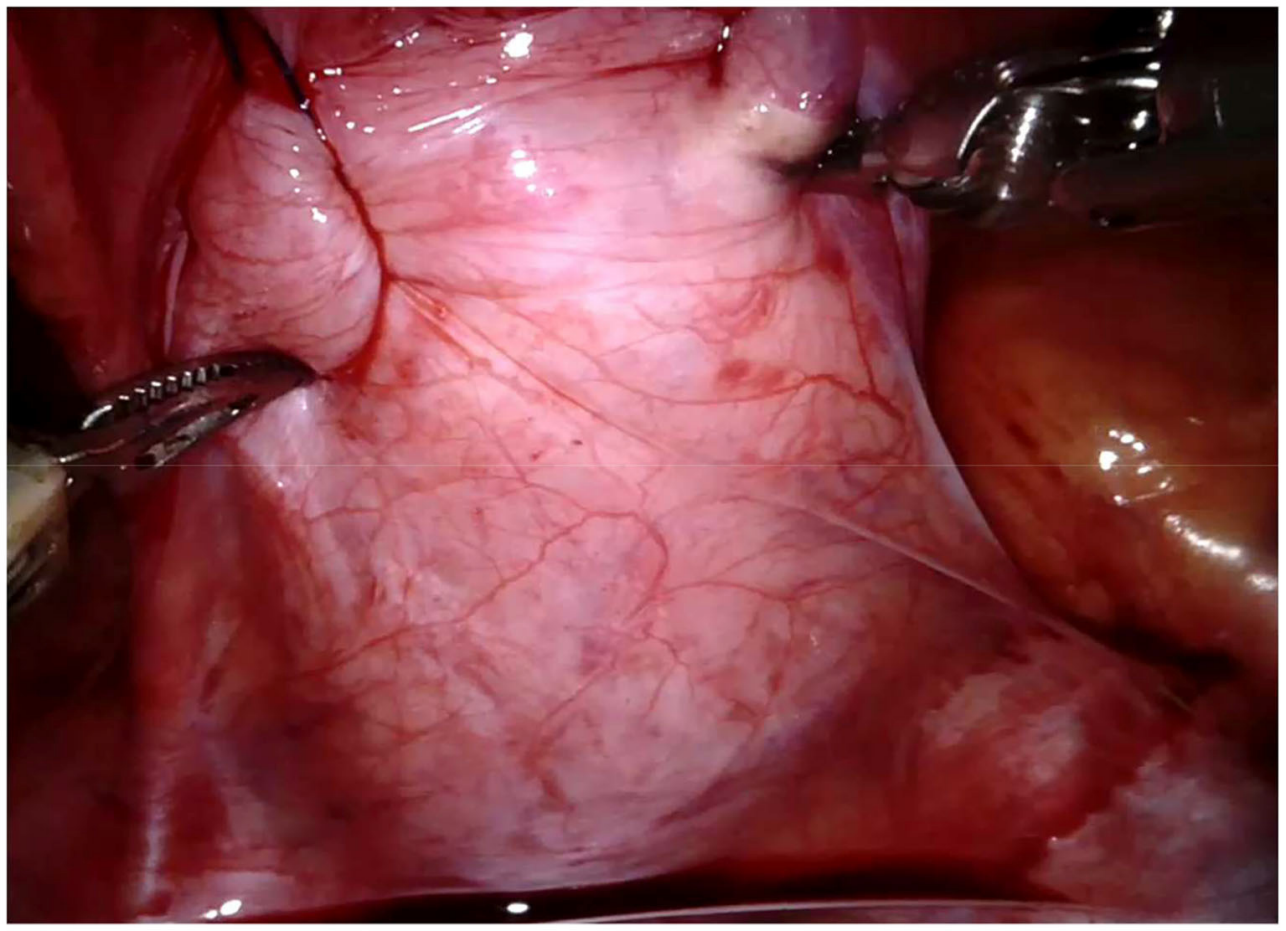
Robotic cystolithotomy technique: bladder suspension.

**Figure 3:**
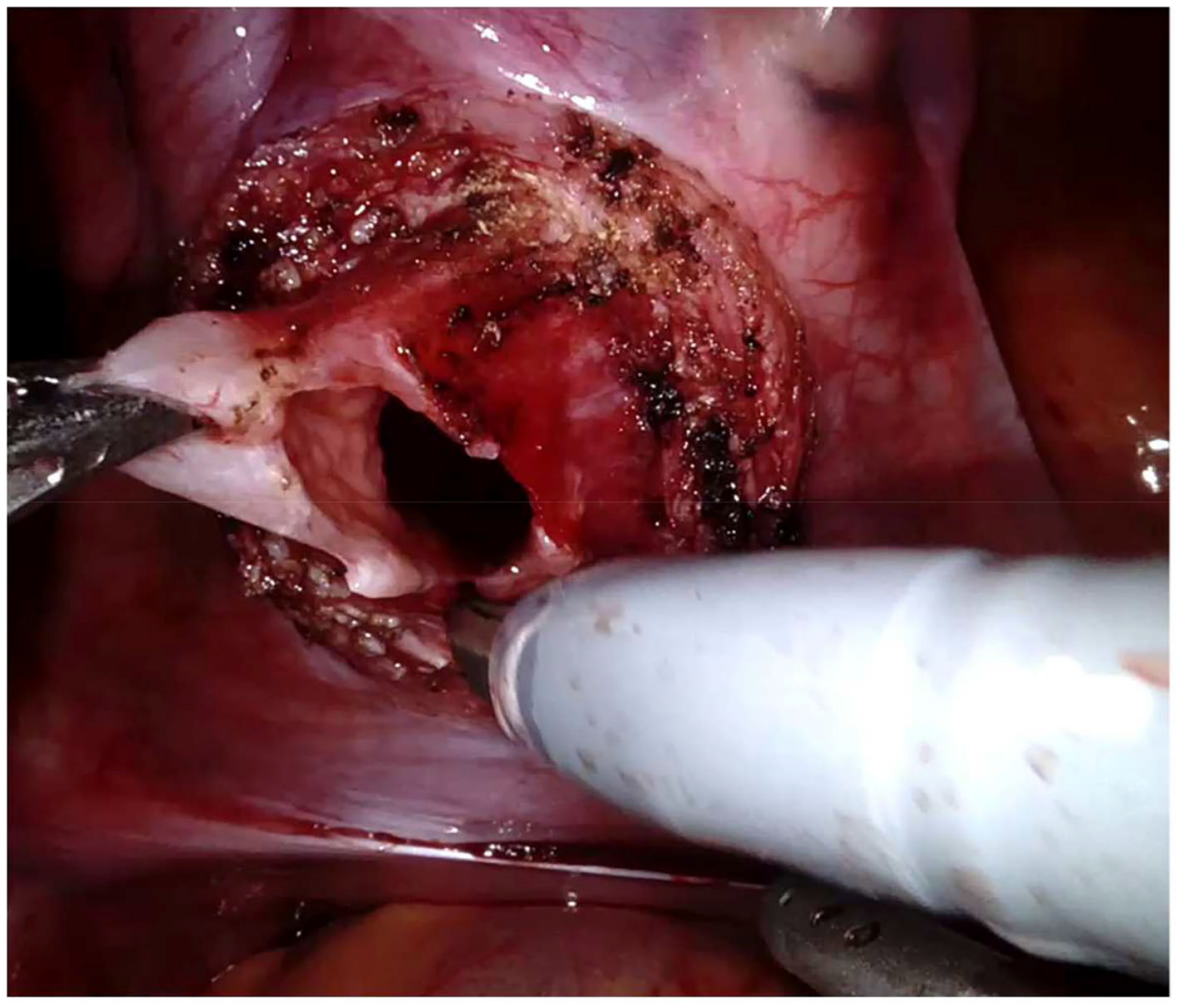
Robotic cystolithotomy technique: bladder opening.

**Figure 4:**
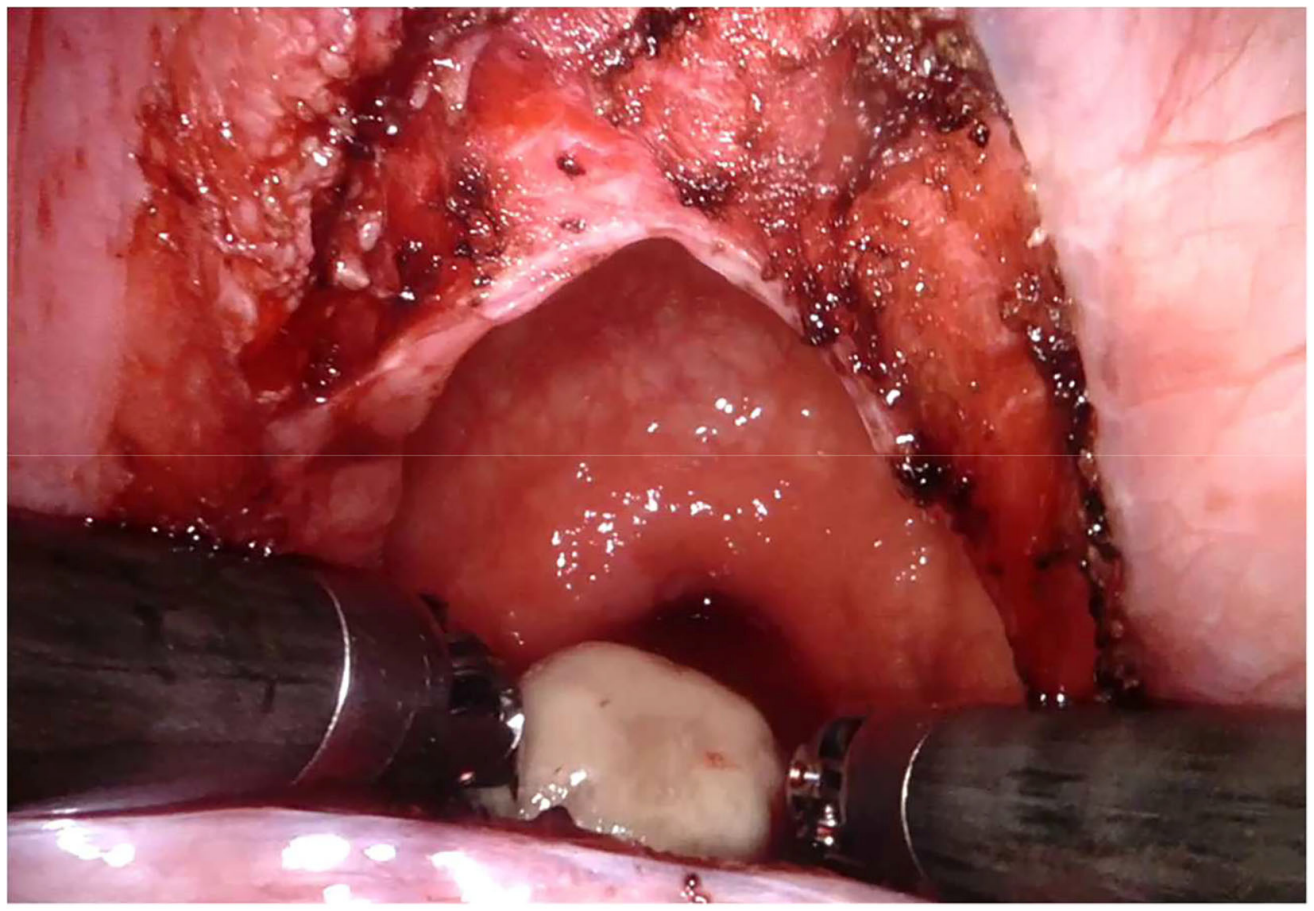
Robotic cystolithotomy technique: stone removal.

Regarding the robotic cystolithotomy, four trocars were placed: one 8-mm robotic port was inserted in the umbilicus for the 30-degree robotic optic and other two 8-mm robotic ports were inserted at 7–9 cm apart from the camera port along the midclavicular line bilaterally for robotic 8-mm instruments (needle holder, scissors, Maryland, and fenestrated forceps). We also adopted an additional 5-mm trocar for the bedside surgeon to introduce and remove the needles, to cut the sutures and to expose or retract tissues. The Robot was then docked over the patient's feet. The bladder was suspended using two stay sutures that were introduced intra-corporeally through the abdominal wall (Figure 2). After filling the bladder with normal saline, a 3.5 cm longitudinal incision of the detrusor muscle was performed in the midline using monopolar scissors, till the mucosa was seen pouting out. The bladder mucosa was also incised and the bladder cavity was inspected (Figure 3). Once visually identified the big stone, it was grasped using the robotic grasper and put into a retrieval bag, that was extracted through the umbilical port at the end of the procedure (Figure 4). The bladder was then flushed with normal saline in order to ensure removal of all stone fragments and the bladder wall was finally reconstructed using a two-layer running 3–0 polyglactin suture, first closing the mucosa and thereafter the detrusor muscle. Trocars' orifices were closed using resorbable sutures. No abdominal drain was placed at the end of the surgery, whereas an indwelling Foley catheter was left into the bladder.

The three methods were compared regarding success rate, operative time, indwelling bladder catheter duration, length of stay (LOS), costs.

Statistical analysis was carried out by using the Statistical Package for Social Sciences (SPSS Inc., Chicago, Illinois, USA), version 13.0. Demographic data were compared using the Student's *t*-test. The categorical variables were compared using χ2 tests. Significance was defined as *p* < 0.05.

## Results

**Figure 5:**
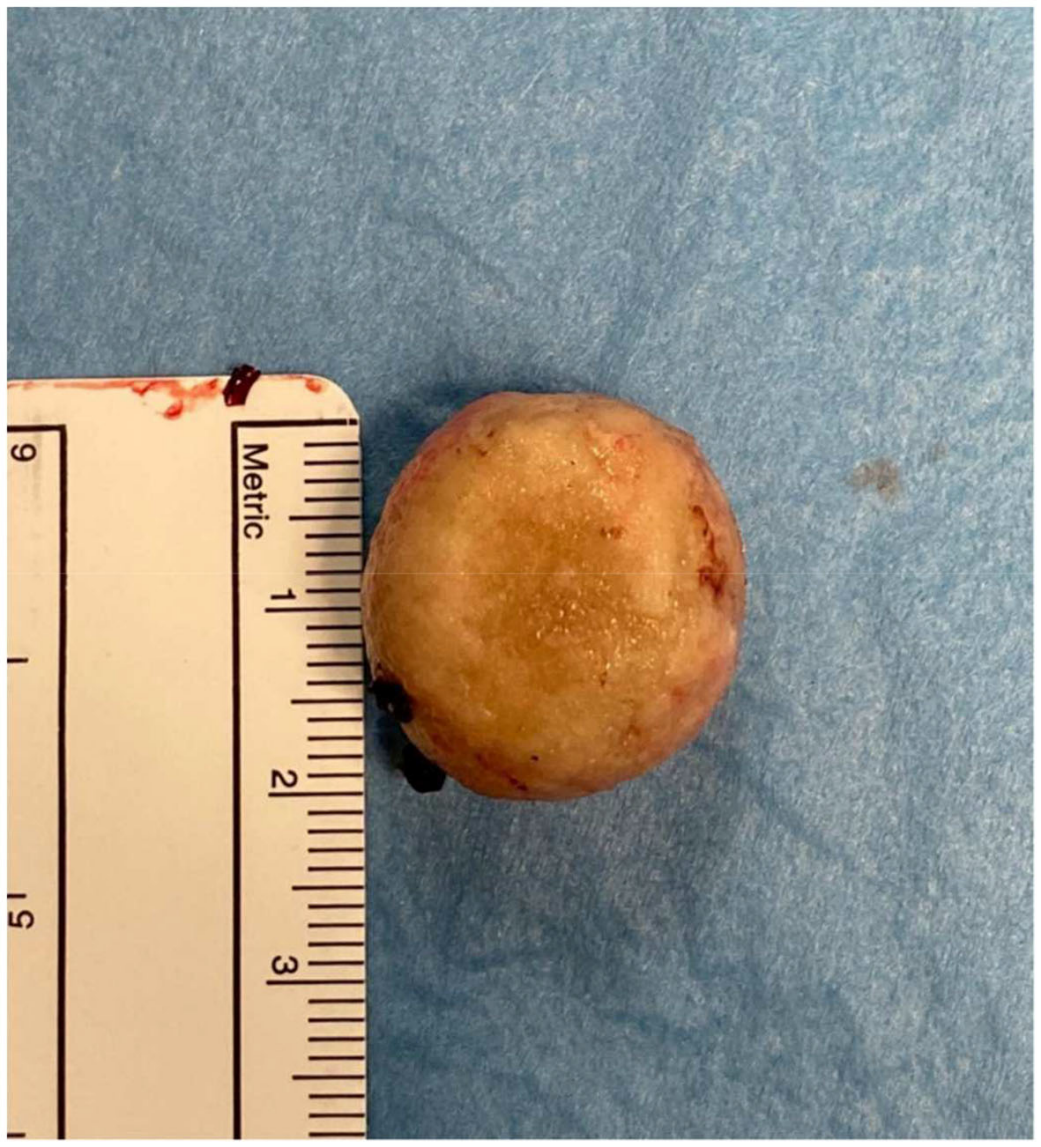
Stone size.

Mean patients' age at the time of diagnosis was 13 years (range 5–18). Regarding the distribution of the origin of the lithiasis, 10/13 patients (76.9%) had primary BS and 3/13 patients (23.1%) had secondary BS. Specifically, two patients had neurogenic bladder dysfunction and performed daily clean intermittent catheterization (CIC) and one patient had previously received endoscopic injection of non-resorbable bulking agent for treatment of vesicoureteral reflux (VUR) in another hospital. In this last patient, the BS were secondary to foreign body reaction due to the bulking agent particles migrated into the bladder. Mean stone size was 18.8 mm (range 7–50) (Figure 5).

The composition of the removed stones was always analyzed after surgery and the patients were referred to pediatric nephrologists for the long-term follow-up. Stone composition was calcium oxalate in 7/13 (53.8%), calcium phosphate in 5/13 (38.5%), and struvite in 1/13 (7.7%).

Patients' characteristics are reported in Table 1.

**Table 1:** Patients' characteristics.

**Patients' characteristics**	
Case number, *n*	13
Gender (male), *n* (%)	8 (61.5%)
Mean age, years (range)	13 (5–18)
Mean weight, Kgs (range)	52.2 (26–77)
**Associated anomalies** Neurogenc Bladder, *n* (%) Previous endoscopic injection for VUR, *n* (%) Other, *n* (%)	2 (15.4%) 1 (7.7%) 0
**Presentation symptoms:** Abdominal pain, *n* (%) Hematuria, *n* (%) Urinary tract infections (UTIs), *n* (%) Asymptomatic, incidental finding, *n* (%)	2 (15.4%) 4 (30.8%) 8 (61.5%) 1 (7.7%)
**Stone origin:** Primary, *n* (%) Secondary, *n* (%) Migratory, *n* (%)	10 (76.9%) 3 (23.1%) 0
**Stone characteristics:** Mean stone size, mm (range) Mean number of stones, *n* (range) Multiple stones present, *n* (%)	18.8 (7–50) 1 (1–3) 4 (30.8%)
**Stone composition:** Calcium oxalate, *n* (%) Calcium phosphate, *n* (%) Struvite, *n* (%)	7 (53.8%) 5 (38.5%) 1 (7.7%)

*VUR, vesicoureteral reflux*.

The stones were removed successfully in all cases. All the procedures were completed using minimally invasive approach without conversions.

Operative time varied between 40 and 90 minutes (mean 66) with no significant difference between the three methods (*p* = 0.8). All the patients had a Foley catheter into the bladder following surgery. Mean indwelling bladder catheter duration was 15.6 h (range 12–72) following robotic cystolithotomy and endourological approach. Conversely, the patients, who received PCCL, kept the catheter longer time than the others (mean 72 h, range 24–96) (*p* = 0.001). The LOS varied between 3 and 7 days (mean 4.7) in patients undergoing robotic and endourological approaches. Patients who underwent PCCL remained hospitalized longer time compared with the other patients, between 7 and 10 days (mean 7.6) (*p* = 0.001).

Mean follow-up length was significantly shorter in patients undergoing robotic cystolithotomy (2.2 years) compared with those undergoing endourology (5.8 years) and PCCL (4.5 years) (*p* = 0.001).

A urinary leak developed postoperatively in one patient of PCCL group (33.3%), who required a prolonged bladder catheterization (Clavien II).

Stone-free rate was 100% and at 1-year follow-up all patients were observed to be stone-free on ultrasound and abdominal x-ray.

We also analyzed the costs of each method. For the authors' hospital, the reimbursement for robotic cystolithotomy was € 9.516, for endourological treatment € 3.507, and for PCCL € 7.775. The endourological approach was the most cost-effective method compared with the other two approaches (*p* = 0.001).

Outcome parameters and comparative analysis between the three methods are reported in Table 2.

**Table 2:** Outcome parameters and comparative analysis.

**Outcome Parameter**	**Robotic cystolithotomy (*n* = 5)**	**Endourology approach (*n* = 5)**	**PCCL (*n* = 3)**	***P*-value**
Mean operative time, minutes (range)	73.8 (60–90)	60 (40–80)	64.2 (55–90)	0.8
Intra-operative complications, *n* (%)	0	0	0	n/a
Conversion, *n* (%)	0	0	0	n/a
Mean indwelling bladder catheter duration, hours (range)	18.6 (12–72)	12.6 (12–60)	72 (24–96)	0.001
Mean length of stay (LOS), days (range)	5.5 (3–7)	3.9 (3–5)	7.6 (7–10)	0.001
Mean follow-up length, years (range)	2.2 (0.2–4)	5.8 (1–7)	4.5 (2–6)	0.001
Stone-free rate, *n* (%)	5 (100)	5 (100)	3 (100)	0.33
Post-operative complications, *n* (%)	0	0	1/3 (33.3%)	0.16
Costs, €	9.516	3.507	7.775	0.001

*PCCL, percutaneous cystolithotomy*.

## Discussion

Although they represent only 5% of all urinary tract stones, bladder stones (BS) are responsible for more than 14% of hospital admissions (1, 2).

BS are more prevalent in males and in developing countries and they have a bimodal age of distribution: incidence peaks at 3 and 60 years (2). BS may cause lower urinary tract symptoms, infections, pain, and hematuria and have been associated with bladder cancer (3).

BS are classified as primary, secondary, and migratory (3). Primary or endemic stones occur in the absence of other urinary tract pathologies, classically seen in children (20). Secondary BS occur in the presence of other urinary tract abnormalities, including bladder outlet obstruction, neurogenic bladder dysfunction, foreign bodies including catheters, bladder diverticula, and bladder augmentation or urinary diversion. Migratory BS form in the upper urinary tract.

However, bladder stones in pediatric population is a rare pathology (3).

Analyzing the international literature, there are several techniques to treat BS using open cystolithotomy, endourology, percutaneous techniques and more recently robotic surgery (21–25).

Strong scientific evidence regarding the optimal approach to BS in the pediatric population is still lacking, but the various endourological approaches have become the mainstay of surgical management of patients presenting with this rare pathology. Open surgery was considered the gold standard treatment of BS in pediatric patients for a long time, offering excellent success rates (26). Al-Marhoon et al. (27) compared open and endourological cystolithotomy (including transurethral and percutaneous approaches) in pediatric patients and showed that all three treatment modalities were very effective with a 100% stone-free rate. However, differences emerged when length of hospital stay (LOS) and complications were compared. LOS was significantly shorter with the endourological approaches (2.6 vs. 4.8 days, *p* < 0.05) but this advantage was mitigated by a greater number of complications (7.4 vs. 0%).

The results of a systematic review and meta-analysis on behalf of the European Association of Urology Urolithiasis Guideline Panel suggested that endoscopic surgery is equally effective as open surgery (21). Endoscopic treatments appeared to have shorter catheterisation time and convalescence compared with open surgery in adults and children (21).

Many urologists would suggest that open cystolithotomy should be performed through a small incision without postoperative catheter drainage, as an outpatient procedure or with an overnight hospitalization (27). This approach would further decrease any advantage that the more complex endourological procedures might offer (27).

In the last few years, transurethral lithotripsy has become an alternative method to open cystolithotomy (8–10). However, the adoption of this approach in the pediatric patients is limited by the narrow caliber of the urethra in children (11). Transurethral extraction with appropriately sized cystoscopic instruments is the treatment of choice if the stone is small and can be completely removed with minimal manipulation and no urethral trauma (27). However, in any other case, perhaps a simple open cystolithotomy may offer the most effective and lowest morbidity patient care. In developing countries, with no advanced methods and equipment, and with many patients unable to pay the costs of less invasive procedures, open surgery was safe, effective, with acceptable hospitalization, excellent patient acceptance, low cost and low morbidity, and provided good stone-free rates (28).

The development of miniaturized instrumentation, associated with increased experience of endourologists with endoscopic procedures, has led to more minimally invasive approaches to BS in pediatric patients.

With the increasing use of percutaneous techniques, a plausible simple solution in children should be percutaneous suprapubic bladder stone removal (PCCL) (13, 14). PCCL has been demonstrated to be adequate and rapid in managing BS in children. However, PCCL has a high complication rate in children (25). For instance, leakage of urine, fistula formation or acute abdomen secondary to intra-peritoneal bladder perforation, have been reported postoperatively. The problem with PCCL is that, using this technique, it's not easy to close the bladder through a 3–4 cm incision and sometimes there are leaks that facilitate formation of a fistula. Moreover, there are complications linked to the technique itself, such as bladder perforation or bleeding (25). At beginning of our experience with PCCL, we experienced a urinary leak in one patient, who required a prolonged bladder catheterization. According to our current protocol, we kept the bladder catheter longer time in patients undergoing PCCL than those undergoing the other procedures, in order to allow a safe closure of the bladder and avoid complications. This protocol obviously required a longer hospitalization but we did not report any other complications, such as urinary leak, fistula formation or other, following PCCL. We adopted this technique to remove big stones until 2016; since 2016, we preferred to adopt robotic surgery.

In the last 5 years, robotic surgery had a huge development, thanks to the 7 degrees of freedom of robotic instruments, the 3D vision for the surgeon and the accuracy of intra-corporeal suturing (29–31). Robotic approach seems to be ideal for BS removal in patients with stones bigger than 15 mm, in patients with narrow urethra or in patients with neurogenic bladder.

The main advantage of robotic cystolithotomy is that it is possible to perform a true minimally invasive procedure and there is no risk of damaging the urethra, that is, reported when you remove the stone fragments through it as it happens using endoscopic approach.

In addition, thanks to the perfect suture of the bladder using robotics instruments, there is no risk of bladder leaks or perforations, as we reported in our series.

However, before starting robotic procedure, it is important to perform a cystoscopy to check the bladder anatomy and the stone location.

Technical key points of the procedure include: fixing the bladder to the abdominal wall with two transabdominal stitches (Figure 2), using an endobag to remove the stone and closing the bladder in two layers.

The greatest limitation of adopting robotic surgery in smaller children is the reduced working space available in such patients, related to the small dimensions of the abdominal cavity and the dilation of the intestinal loops. An important trick is to perform an intestinal preparation with simethicone for 1 week prior to surgery, and enema and minimal-residue diet the day before surgery. As we already reported in laparoscopic surgery (32), we found that the intestinal preparation was also helpful in robotics, especially in children weighing <15 Kg. In fact, the main benefit of intestinal preparation was that deflated bowel loops falled away from the bladder very easily and provided a better exposition of the operative field and a larger working space, by keeping a reduced Trendelenburg angle (10°) of the operating table and the insufflation pressure as low as possible (average 12 mmHg). This allowed a successful application of robotic surgery also in smaller children, who are particularly sensible to the effects of pneumoperitoneum.

The main disadvantages of robotics remain the high costs of the procedure. We analyzed the costs of each method and we found that the endourological approach was the most cost-effective method compared with the other two approaches (*p* = 0.001). Regarding the availability of each technique, robotic cystolithotomy obviously required the use of the Robot, which may not be available in all pediatric centers. Endourology required a dedicated pediatric miniaturized instrumentation whereas PCCL with MIS suprapubic approach adopted a common operative laparoscope, that is usually available in each surgical unit with high volume MIS activity.

It is important to remember that urolithiasis is a complex pathology; therefore, these patients must be followed in pre- and post-operative period by a multi-disciplinary team of pediatric nephrologists and pediatric urologists.

Major limitations of the study include the retrospective nature, the restricted number of cases treated and the short follow-up period for the patients, who were treated more recently. However, we should also consider that the incidence of this condition is low in the pediatric population.

As a consequence, our study indicates that further research, comparing the benefits and harms of treatments for BS in children, is required; particularly comparing the different minimally invasive treatments which should stratify patients by stone size and characteristics (including age), as well as define and robustly measure outcomes.

In conclusion, minimally invasive management of bladder stones in children was safe and effective. Endourological management was the most cost-effective method, allowing a shorter hospital stay compared with the other procedures but it was mainly indicated for smaller stones with a diameter < 10 mm. Based upon our preliminary results, robotic surgery seemed to be a feasible treatment option for bladder stones larger than 15–20mm. It allowed to remove the big stones without crushing them with a safe and easy closure of the bladder wall thanks to the easy suturing provided by the Robot technology.

Further research is required to clarify the efficacy of minimally invasive treatments for larger stones in young children. The choice of the optimal method to achieve stone clearance should be tailored to the parameters of the single patient (anatomy, stone burden) as well as the availability of equipment and the surgeon's experience.

## Data Availability Statement

The raw data supporting the conclusions of this article will be made available by the authors, without undue reservation.

## Author Contributions

CE contributed conception and design of the study and wrote the first draft of the manuscript. GA, LM, MC, FD, VC, MC, FC, and ME organized the database and wrote sections of the manuscript. All authors contributed to manuscript revision, read and approved the submitted version.

## Conflict of Interest

The authors declare that the research was conducted in the absence of any commercial or financial relationships that could be construed as a potential conflict of interest.
